# Statin therapy in chronic viral hepatitis: a systematic review and meta-analysis of nine studies with 195,602 participants

**DOI:** 10.1080/07853890.2021.1956686

**Published:** 2021-07-23

**Authors:** Amir Vahedian-Azimi, Sajad Shojaie, Maciej Banach, Farshad Heidari, Arrigo F. G. Cicero, Masoum Khoshfetrat, Tannaz Jamialahmadi, Amirhossein Sahebkar

**Affiliations:** a Trauma Research Center, Nursing Faculty, Baqiyatallah University of Medical Sciences, Tehran, Iran; b Basic and Molecular Epidemiology of Gastrointestinal Disorders Research Center, Research Institute for Gastroenterology and Liver Diseases, Shahid Beheshti University of Medical Sciences, Tehran, Iran; c Department of Hypertension, Chair of Nephrology and Hypertension, Medical University of Lodz, Lodz, Poland; d Cardiovascular Research Centre, University of Zielona Gora, Zielona Gora, Poland; e Nursing Care Research Center (NCRC), School of Nursing and Midwifery, Iran University of Medical Sciences, Tehran, Iran; f Atherosclerosis Research Unit, Medical and Surgical Sciences Department, Sant’Orsola-Malpighi Hospital, University of Bologna, Bologna, Italy; g Department of Anesthesiology and Critical Care, Khatamolanbia Hospital, Zahedan University of Medical Sciences, Zahedan, Iran; h Department of Food Science and Technology, Quchan Branch, Islamic Azad University, Quchan, Iran; i Department of Nutrition, Faculty of Medicine, Mashhad University of Medical Sciences, Mashhad, Iran; j Biotechnology Research Center, Pharmaceutical Technology Institute, Mashhad University of Medical Sciences, Mashhad, Iran; k Applied Biomedical Research Center, Mashhad University of Medical Sciences, Mashhad, Iran; l School of Medicine, The University of Western Australia, Perth, Australia; m School of Pharmacy, Mashhad University of Medical Sciences, Mashhad, Iran

**Keywords:** Statins, liver, hepatitis, fibrosis, prognosis

## Abstract

**Background:**

Conflicting data suggest that statins could cause chronic liver disease in certain group of patients, while improving prognosis in those with chronic viral hepatitis (CVH).

**Purpose:**

To quantify the potential protective role of statins on some main liver-related health outcomes in clinical studies on CVH patients.

**Data Sources:** The search strategy was explored by a medical librarian using bibliographic databases, from January 2015 to April 2020.

**Data synthesis:** The results showed no significant difference in the risk of mortality between statin users and non-users in the overall analysis. However, the risk of mortality significantly reduced by 39% in statin users who were followed for more than three years. Moreover, the risk of HCC, fibrosis, and cirrhosis in those on statins decreased by 53%, 45% and 41%, respectively. Although ALT and AST reduced slightly following statin therapy, this reduction was not statistically significant.

**Limitations:**

A significant heterogeneity among studies was observed, resulting from differences in clinical characteristics between statin users and non-users, study designs, population samples, diseases stage, comorbidities, and confounding covariates.

**Conclusion:**

Not only long-term treatment with statins seems to be safe in patients affected by hepatitis, but also it significantly improves their prognosis.

## Introduction

3-hydroxy-3-methylglutaryl coenzyme A (HMG-CoA) reductase inhibitors (statins) are a cornerstone of hypercholesterolaemia treatment and cardiovascular disease prevention [1]. Statins are generally safe, with the most commonly reported side effects being an increase in creatine phosphokinase and/or myalgias [2–5]. These side effects have been proposed to be due to a negative impact of statins on coenzyme Q10 synthesis [6,7], over-expression of the muscle-specific ubiquitin-proteasome system as the major non-lysosomal intracellular protein degradation system [8], and genetic mechanisms [9]. However, a primary concern of statin-related side effects is liver toxicity, which is a major cause of statin treatment interruption [10]. Its pathogenic mechanism is not well understood. Some drug-induced liver injuries (DILI) with autoimmune features have been related to statin use [11]. In most cases, it is characterised by a transient elevation less than three times the normal upper limit of liver transaminases which subsides spontaneously with treatment discontinuation [12]; in some other cases, it is characterised by an acute form of hepatitis with a significant rise in transaminases leading to hepatic failure [13,14]. The risk of statin-related liver toxicity seems to be higher in the elderly, especially when it is accompanied by alcohol consumption and hepatitis [15]. These observations could reduce the physician’s confidence in treating patients affected by chronic liver diseases with statins, even if the number needed to treat (NNT) to prevent one acute cardiovascular event with statins as monotherapy or in combination with other cholesterol-lowering medications is between 3 and 61, depending on the risk and LDL-C [16], while the number needed to harm (NNH) for a statin-related acute liver injury is estimated to be 1:1,500,000. However, most patients tolerate statin treatment, particularly those with HCV-HIV co-infection [17]. Moreover, increasing evidence that suggests statins could also exert some hepatoprotective effects, which have been recently investigated in different preclinical models of liver cell peroxidation, viral infection, inflammation and fibrosis [18]. Conflicting results also suggest that statins could positively impact the prognosis of some chronic liver diseases and related complications in humans [19]. In this context, the aim of our meta-analysis was to quantify the potential protective role of statins on some main liver-related health outcomes in large prospective human studies.

## Methods

This systematic review was conducted in accordance with the Preferred Reporting Items for Systematic Reviews and Meta-Analyses (PRISMA) guidelines. Ethical approval was obtained from the research ethics committee of Baqiyatallah University of Medical Sciences with the ethics code of IR.BMSU.REC.1399.009 in 2020-03-26.

### Search strategy

A computer-based literature search was conducted in April 2020 using Web of Science (ISI), PubMed, Embase, Scopus, ProQuest, Ovid, EBSCO, and CINAHL for eligible articles, until 2 April 2020, with no restrictions on language or publication date. The reference list of articles was reviewed using forward and backward citation tracking to identify other eligible documents.

The PICO question (Population, Intervention, Comparison and Outcome) was formulated as follows: Population: humans – Intervention: statin therapy – Comparison: subjects without statin therapy – Outcomes: chronic viral hepatitis complications (mortality, hepatocellular carcinoma (HCC), fibrosis, cirrhosis). “Does a relationship exist between statin therapy and improved complications in patients with chronic viral hepatitis?”

The following MeSH and non-MeSH search terms were used to encompass the effects of every type of statin therapy on chronic viral hepatitis complications: Statin OR “HMG CoA reductase inhibitor” OR lovastatin OR fluvastatin OR pravastatin OR rosuvastatin OR pitavastatin OR atorvastatin OR simvastatin OR cerivastatin OR lipitor OR lescol OR lescol AND xl OR mevacor OR altoprev OR pravachol OR crestor OR zocor OR livalo AND “Viral infection” OR viral OR virus OR virol*.

To comprehensively search all articles related to the effect of statins and not to miss an outcome, we followed the search without considering a specific outcome. After finding all the articles, we concluded that only four outcomes, including mortality, HCC, fibrosis and cirrhosis, can be meta-analysed, and so the study continued with a focus on these four outcomes.

### Inclusion and exclusion criteria

The inclusion criteria were as follows:

- Observational studies (case-control studies and cohort studies) and randomised clinical trials evaluating the impact of statins in patients with chronic viral hepatitis. Both prospective and retrospective studies were included.
- Outcome measures that included mortality rate, development of HCC, cirrhosis, and fibrosis.
- Clear definition and diagnosis of HCC, cirrhosis and fibrosis; can be diagnosed by imaging test such as computed tomography (CT), abdominal ultrasound or magnetic resonance imaging (MRI) or via a needle biopsy of the liver

The exclusion criteria were as follows:

- Clinical case reports, literature reviews, editorials, animal studies, and studies involving in vitro experiments were not included.
- Studies not including statin nonusers’ subjects.

### Data extraction

Data extraction was performed in parallel by two authors independently. The following information was obtained: authors' name, year of publication, sample size, subjects, reported rate, setting, study design, result, and conclusion. Furthermore, the main outcomes were summarised and included. Any differences in opinion in the screening process, data extraction, and analysis were resolved through a re-examining the study and further discussion. In case of any disagreement, the reviewers could consult a third reviewer. The extracted data is displayed in Tables S1–S4.

### Quality assessment

The methodological quality assessment of studies was performed independently by two authors using the Newcastle-Ottawa Scale (NOS) for Cohort studies and the Jadad scale for randomised trial studies. NOS scale was developed to assess the quality of nonrandomized studies with its design, content, and ease of use directed to incorporate the quality assessments in the interpretation of meta-analytic results. In this scale, a study is judged on three broad perspectives: the selection of the study groups; the comparability of the groups; and the ascertainment of either the exposure or outcome of interest for case-control or cohort studies, respectively [20]. A star rating system is used to indicate the quality of a study, with a maximum of nine stars [21]. The Jadad scale was used to perform a quality assessment of the included studies. The Jadad scale is a three‐item, validated, and reliable scoring tool for randomised controlled trials (RCTs). The scale focuses on randomisation, blinding, and withdrawals/dropouts. Studies can be given a total score of 0 to 5, with 5 being the most ideal score and a score of 3 or greater considered to be high quality [22]. Disagreements about inclusion criteria, data extraction, and quality assessment were resolved by consensus. The quality assessment of studies is displayed in Tables S5 and S6.

### Data synthesis and statistical analysis

We applied fix-effects or random-effects meta-analyses with inverse variance (IV) weighting to calculate pooled estimates and confidence intervals (95% CIs). We calculated the odds ratios (OR) and 95% CIs for the target community of mortality, outcomes of hepatitis and HCC, fibrosis, and cirrhosis outcomes. We also estimated the pooled effect size as weighted mean difference (WMD) for aspartate aminotransferase (AST) and alanine aminotransferase (ALT) levels. The median and interquartile range were converted to mean and standard deviation based on the method described by McGrath *et al.* [23]. The results were obtained in the subgroup and overall analysis and illustrated by the Forest plots. Heterogeneities were assessed using *I*^2^ measure and Cochrane’s Q statistic within or between study design [24]. We performed subgroup analysis to find the potential source of the heterogeneity and to investigate the effect of each predefined criteria. Assessment of publication bias was evaluated by Egger’s test [25]. Radial (Galbraith) and Funnel plots were illustrated to assess heterogeneity and publication bias. Furthermore, we conducted some analysis to identify outlying studies *via* techniques [26] based on the Leave-One-Out method to detect studies that influence the overall estimate of our meta-analysis. The influence diagnostics and Baujat [27] plot showed a variety of influence and outlier analyses. In two forest plots, we observed the pooled effect recalculated, with one study omitted each time. All statistical analysis was carried out using STATA, version 16.0 (Stata Corp, College Station, TX) and R version 3.6.3.

## Results

### Search outcomes and study characteristics

**Figure 1.**
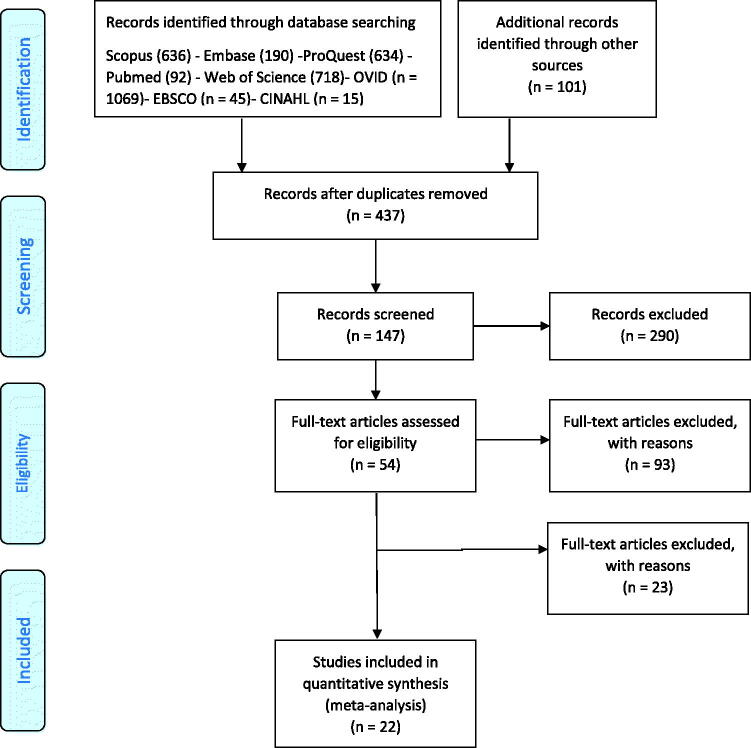
Flowchart of study selection process.

Literature search, data extraction, and general description of included studies were carried out by two independent researchers (FHB and AVA). Total of 3399 articles was searched from Scopus (*n* = 636), Embase (*n* = 190), ProQuest (*n* = 634), PubMed (*n* = 92), Web of Science (*n* = 718), Ovid (*n* = 1059), EBSCO (*n* = 45), and CINAHL (*n* = 15). In addition, 102 studies identified through other sources were added and finally the total identified studies reached 3,501. The full list of files was reviewed, duplicated studies were excluded and 437 records were retained. After titles and abstracts screening, relevant studies were selected for full text evaluation and 290 studies were excluded. The full text of the 147 remaining studies assessed for eligibility and 54 studies remained in the next step. Finally, the 22 studies according to eligibility criteria were included in this study. The study selection process is shown in Figure 1.

We identified 363,174 participants from 22 studies in this meta-analysis. A total of 298,198 subjects entered the study after the elimination of overlapping studies. According to our primary (mortality) and secondary outcomes (HCC, fibrosis and cirrhosis); nine studies with 195,602 subjects were analysed to target the effect of statin on mortality among patients with CVH. In addition, six studies were assessed to determine the effect of statin consumption on the risk of HCC (including 72,690 patients), two studies on the risk of fibrosis (including 9678 patients), and two studies on the risk of cirrhosis (including 85,205 patients). All these studies included patients with positive hepatitis B or positive hepatitis C viruses. Progression of liver fibrosis as estimated by the Fibrosis-4 (FIB-4) index, and development of cirrhosis, defined by a FIB-4 score greater than 3.5 (age(years) × AST(U/L)/platelet (PLT) (109/L) × √ALT(U/L)) [28]. The characteristics of included studies were generally described in the Supplementary material files, Tables S1–S4. Of the nine studies reviewed for mortality, five were from Asian countries (Hong Kong and Taiwan each had two studies and one from Korea) and the rest from the United States (three studies) and Sweden (one study).

### Main outcome: mortality

**Figure 2.**
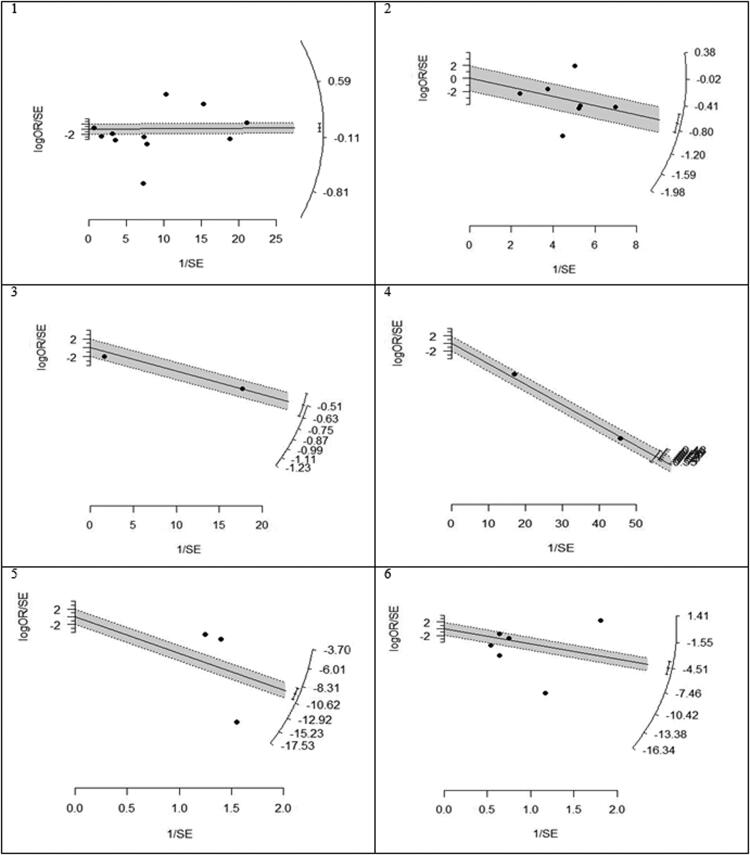
Radial plots to determine heterogeneity between studies design. 1. Mortality, 2. Hepatocellular carcinoma (HCC), 3. Fibrosis, 4. Cirrhosis, 5. AST, 6. ALT.

**Figure 3.**
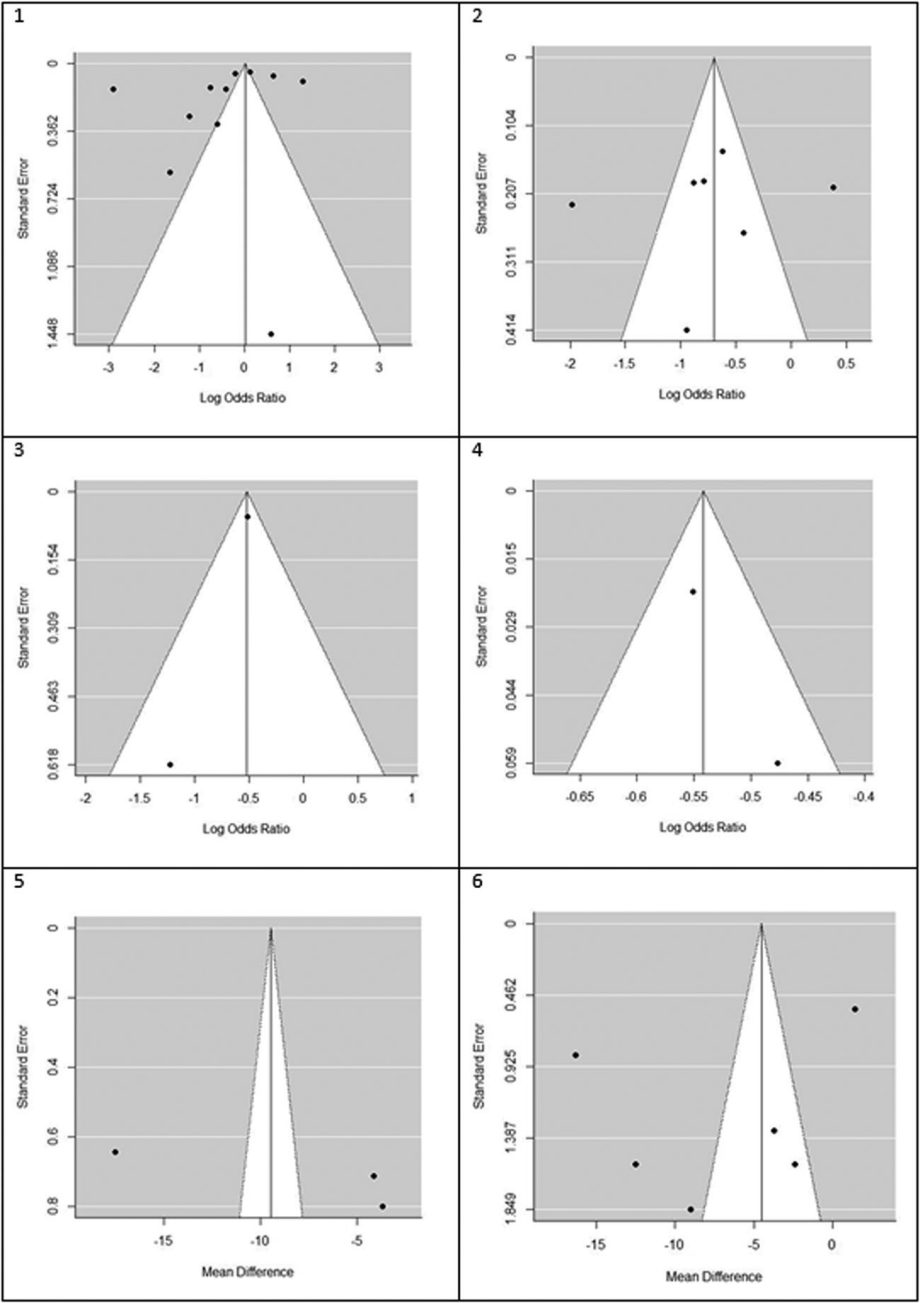
Funnel plots for detecting publication bias of studies. 1. Mortality, 2. Hepatocellular carcinoma (HCC), 3. Fibrosis, 4. Cirrhosis, 5. AST, 6. ALT.

We found that statin use reduced mortality but not statistically significant in the overall analysis (OR (95% CI) = 0.61 (0.35, 1.06), *p* = .082). Heterogeneity between study designs was obtained in the radial plot (*I*^2^ = 98.8%, *p* < .001) (Figure 2). While Egger’s test confirmed no publication bias (*p* = .329) (Figure 3).

However, in one subgroup analysis, different mortality rates were seen in different countries. Statin users had lower mortality rates than non-users in Korea (OR (95% CI) = 0.19 (0.06, 0.60), *p* < .001), while the results were reversed in Hong Kong (OR (95% CI) =2.62 (1.37, 4.98), *p* = .003). However, there was no effect in Taiwan (OR (95% CI) = 0.59 (0.27, 1.30), *p* = .242) and the United States (OR (95% CI) = 0.27 (0.04, 1.73), *p* = .166) (Figure 4). In another subgroup analysis, the effects of statins on mortality rate in Asian and non-Asian subgroups were examined. Although statin reduced mortality in the non-Asian region, it was not statistically significant (OR (95% CI) = 0.40 (0.13, 1.25), *p* = .088) (Figure 5).

Figure 4.Forrest plots of outcomes. 1. Mortality, 2. Hepatocellular carcinoma (HCC), 3. Fibrosis, 4. Cirrhosis, 5. AST, 6. ALT.

**Figure:**
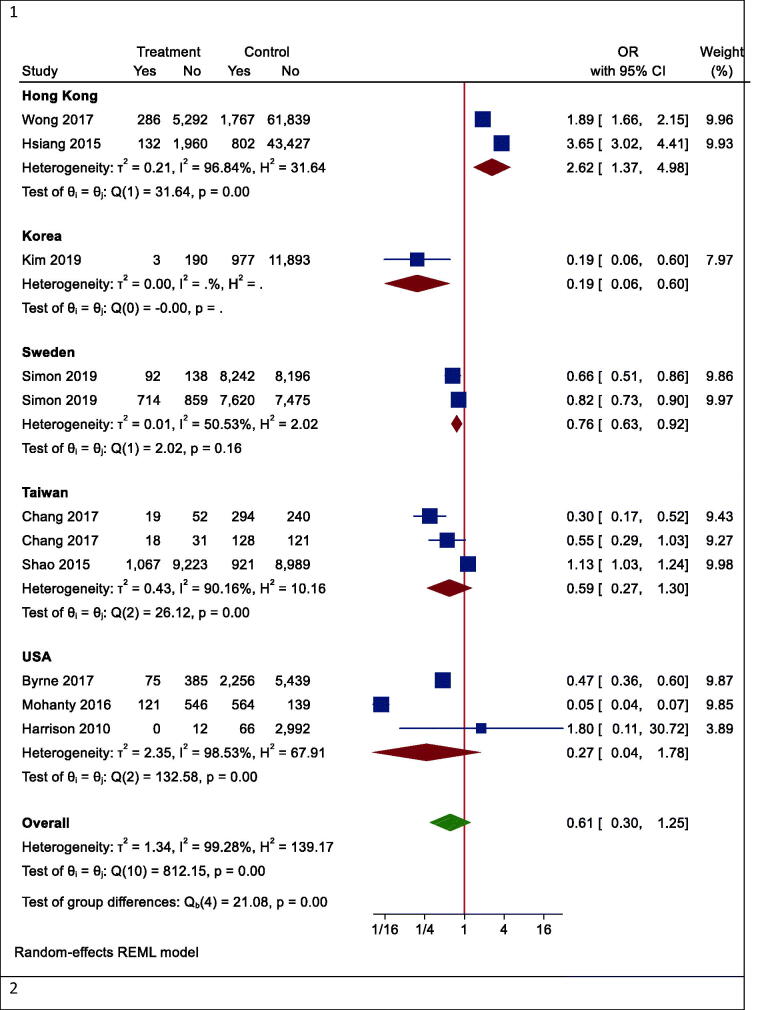

**Figure:**
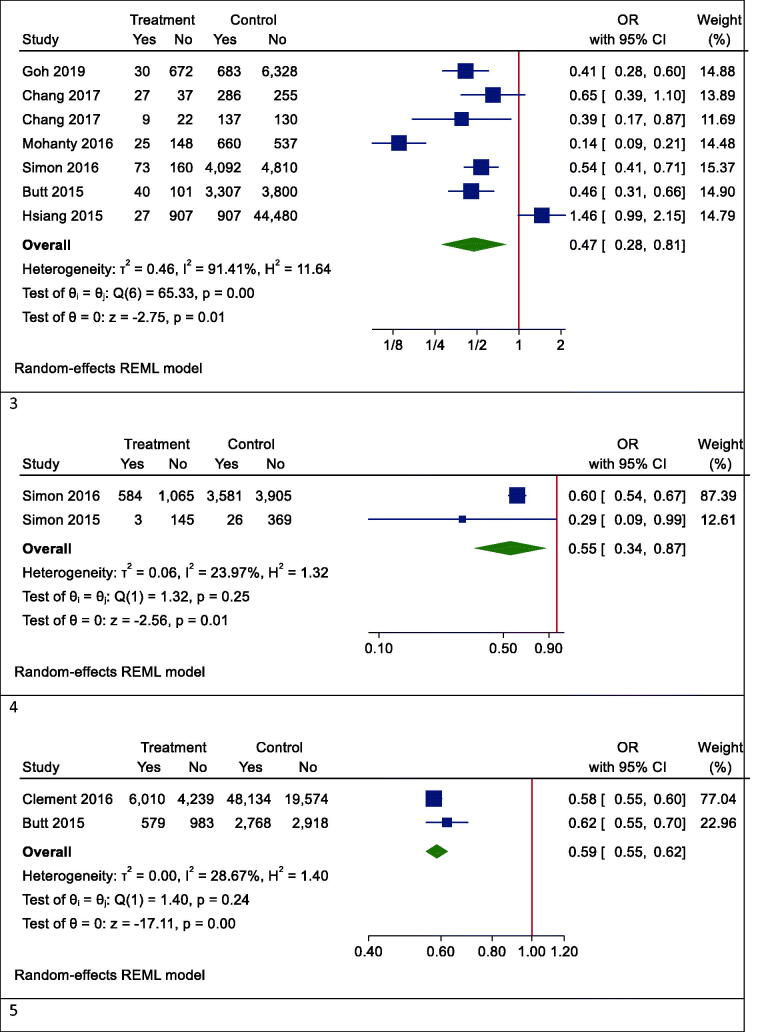

**Figure:**
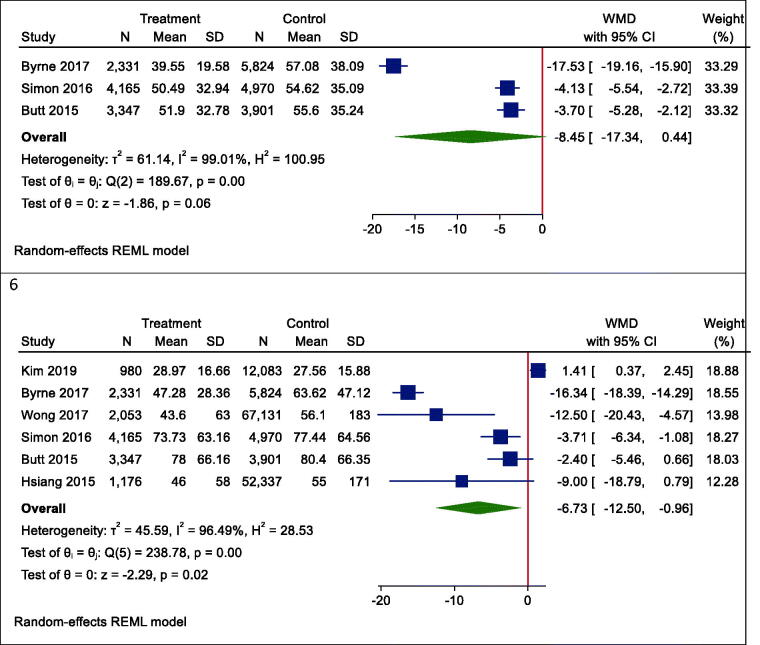

Figure 5.Forrest plots of mortality. 1. Mortality in Asian and non-Asian countries. 2. Mortality in the overall analysis. 3. Mortality in people with other specific medical illnesses. 4. Mortality in patients with different types of hepatitis. 5. Mortality in patients with hepatocellular carcinoma having different types of hepatitis. 6. Mortality rate based on the follow-up period.

**Figure:**
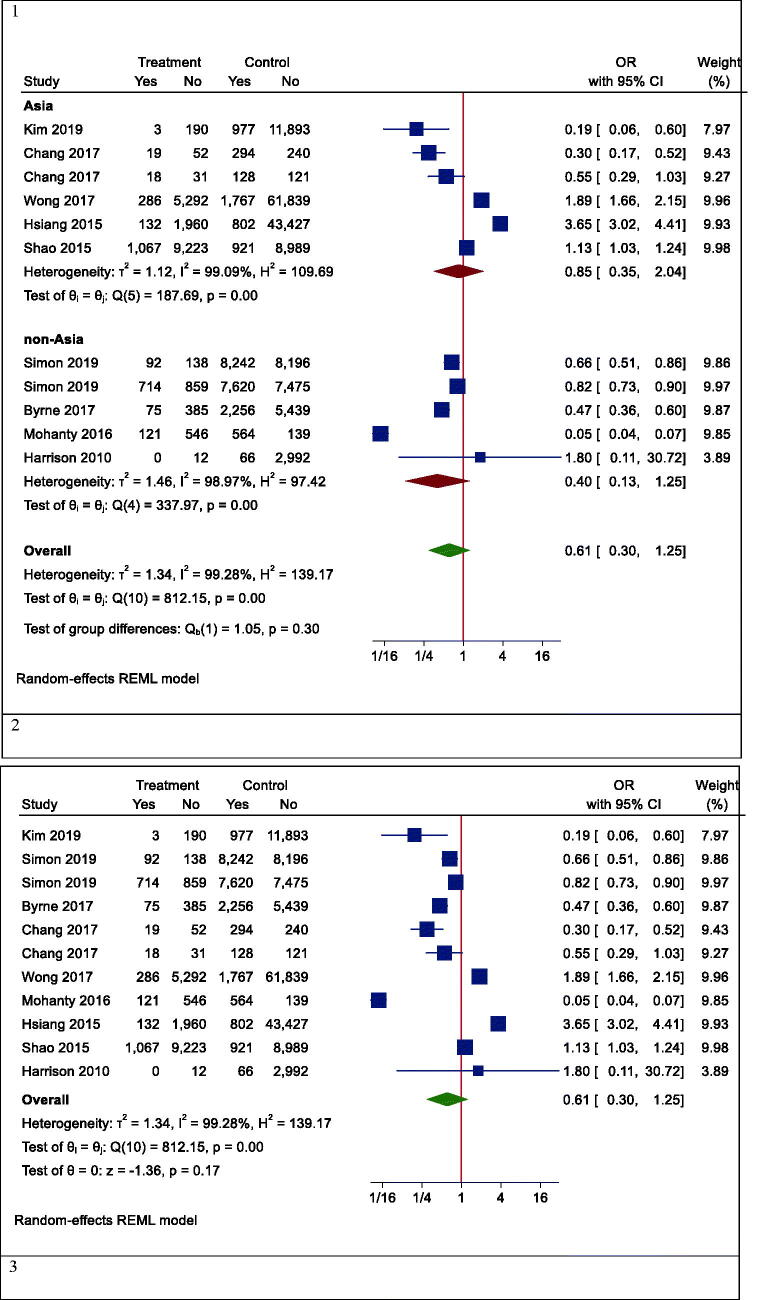

**Figure:**
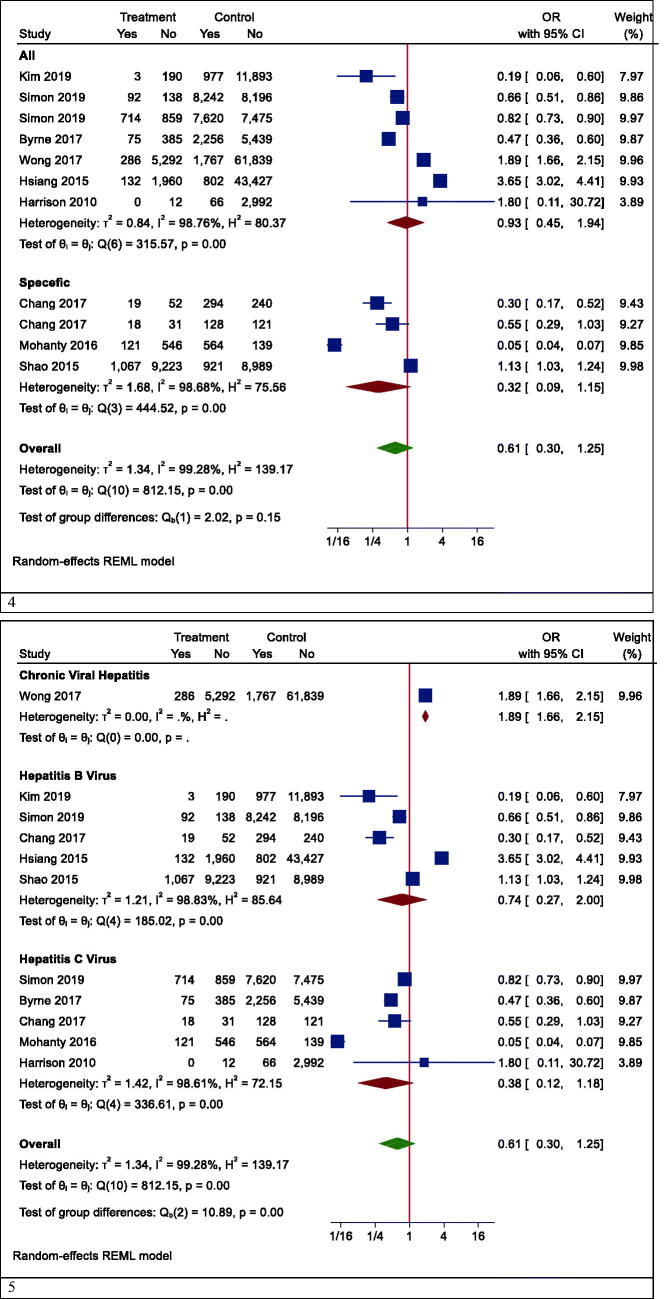

**Figure:**
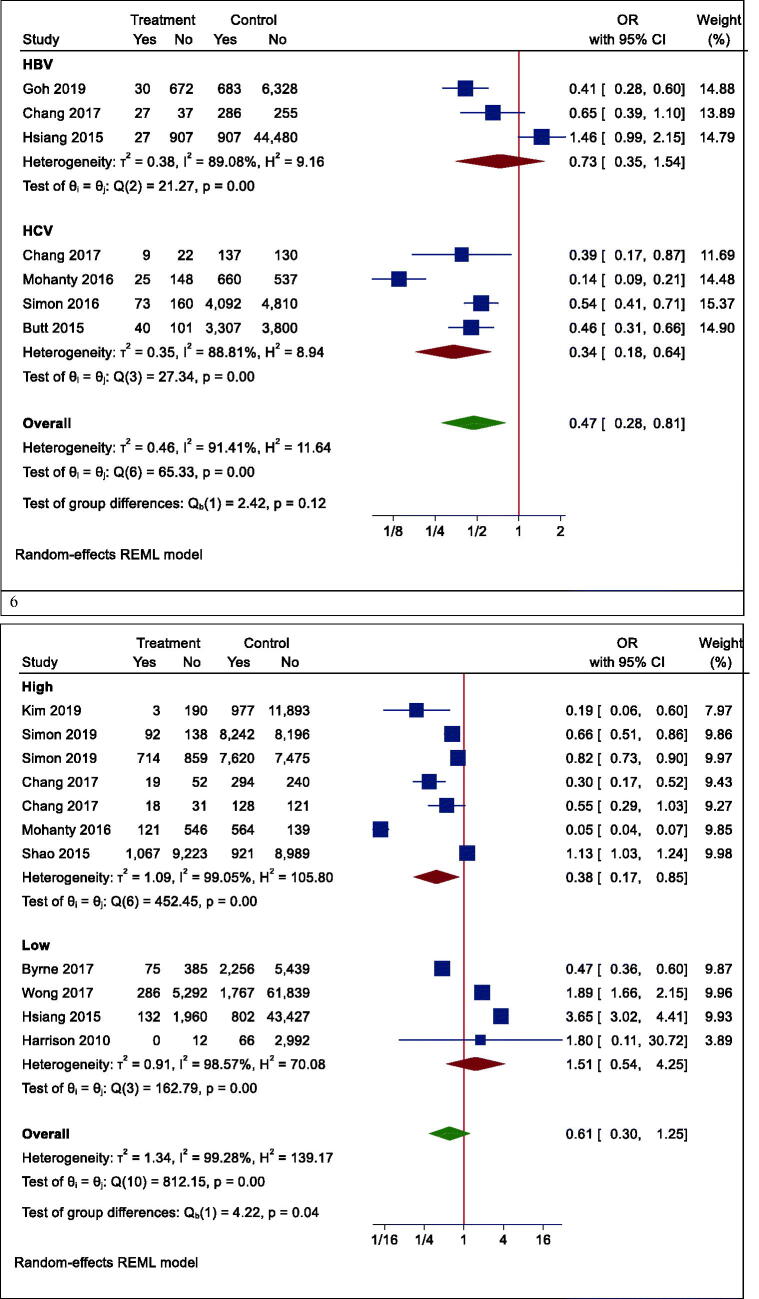

Although the risk of mortality was lower in people with other specific medical illnesses (such as HCC or cirrhosis) who took statins, it was not significant due to the large confidence interval (OR (95% CI) = 0.32 (0.09, 1.15), *p* = .210) (Figure 5). Besides, statins did not significantly reduce mortality in patients with positive hepatitis B or C virus (HBV OR (95% CI) = 0.74 (0.27, 2.00), *p* = .496; HCV OR (95% CI) = 0.38 (0.12, 1.18), *p* = .124) (Figure 5). Moreover, the results of studies were analysed with more than three years of follow-up indicated a significant reduction in all-cause mortality (OR (95% CI) = 0.38 (0.17, 0.85), *p* = .006). In studies with shorter follow-up, however, the insignificant opposite effect was observed (OR (95% CI) = 1.51 (0.54, 4.25), *p* = .379) (Figure 5).

### Influential analysis

Based on the results obtained from the Leave-One-Out method (Figure S1), only Hsiang's study was identified as outlying. Also, according to the right side of the Baujat plot (Figure S2), this study overly contributed to the heterogeneity of the meta-analysis. Furthermore, in these forest plots, ordered by heterogeneity and effect size (small to high), we saw the smallest effect size and I^2^ measure by omitting the study of Hsiang et al. (Figure S3). Our findings demonstrated that this study was probably an outlier, which may distort the effect size estimate, as well as its precision.

### Secondary outcome: HCC

Our results proved that the pooling effect size resulted in a reduction of HCC due to statin use, significantly (OR (95% CI) = 0.47 (0.28, 0.81), *p* = .005) (Figure 4). Heterogeneity between study designs was obtained in the Radial plot (*I*^2^ = 90.8%, *p* < .001) (Figure 2). In addition, there was no publication bias according to Egger’s test (*p* = .750) (Figure 3).

### Secondary outcome: fibrosis

The results for the fibrosis outcome displayed a significant results. In fact, statin consumption decreased fibrosis in the studies (OR (95% CI) = 0.55 (0.34, 0.87), *p* < .001) (Figure 4). Heterogeneity and publication bias were illustrated in the Radial plot and Funnel plot (Figures 2 and 3).

### Secondary outcome: cirrhosis

The results for the cirrhosis outcome showed a significant results. In fact, statin consumption decreased cirrhosis in the studies (OR (95% CI) = 0.59 (0.55, 0.62), *p* < .001) (Figure 4). Heterogeneity and publication bias were illustrated in the radial plot and funnel plot (Figures 2 and 3).

### Secondary outcome: aspartate aminotransferase

According to our meta-analysis with WMD as the summary estimate, a borderline non-significant reduction of AST levels in statin recipients (WMD (95% CI) = −8.45 (−17.34, 0.44), *p* = .075) was noted (Figure 4). Heterogeneity was found in the radial plot (*I*^2^ = 99.2%, *p* < .001) (Figure 2), while Egger’s test confirmed that publication bias was absent (*p* = .340) (Figure 3).

### Secondary outcome: alanine aminotransferase

With respect to ALT, a borderline non-significant reduction in statin recipients (WMD (95% CI) = −6.37 (−12.50, −0.96), *p* = .055) was found (Figure 4). Heterogeneity was found in the radial plot (*I*^2^ = 98.5%, *p* < .001) (Figure 2), while Egger’s test confirmed that publication bias is absent (*p* = .382) (Figure 3).

## Discussion

This study was conducted in a large sample size to comprehensively evaluate the efficacy and safety of statins on liver diseases and in patients with CVH. This issue has been a matter of large discussion in the last decades and is still the reason for the many cases of discontinuation of statin therapy. In the present meta-analysis, 22 studies were finally included for data pooling and synthesis, of which nine studies (195,602 subjects) were analysed to target the effect of statin on mortality in patients with chronic viral hepatitis. In addition, other studies were enrolled in the study to evaluate the effect of statin on HCC, fibrosis, cirrhosis, and AST and ALT levels. The results showed no significant difference in the risk of mortality between statin users and non-users in the overall analysis. However, the risk of mortality was significantly reduced by 39% in statin users who were followed by more than three years compared to those with less than three years of follow-up. Moreover, the risk of HCC, fibrosis and cirrhosis in statin users decreased by 53%, 45% and 41%, respectively. Although the levels of ALT and AST were reduced slightly following statin therapy, this reduction was not significant.

Our findings are consistent with some previous studies; Gu et al. [29] in a meta-analysis showed that long-term treatment with statins significantly lowered mortality rate by 22% and incidence of liver cancer by 25% in patients with chronic liver diseases. In a meta-analysis by Ma et al. [30], 10 observational studies involving 123,445 patients were included, and the results showed that statin use was associated with a significantly reduced risk of virus-related cirrhosis and decompensation. Data derived from three large randomised clinical trials and 10 cohort studies by Kim et al. [31]. showed that statin use was associated with a 46% lower risk of hepatic decompensation and a 46% lower mortality rate in patients with cirrhosis. In the randomised clinical trials, statin use was associated with a 27% lower risk of variceal bleeding or progression of portal hypertension. In addition, a meta-analysis of 16 homogeneous studies by Zheng et al. [32] showed that in 12,791 chronic hepatitis C (CHC) patients who received statins as an adjuvant to general antiviral therapy, the sustained virological response (SVR) rate increased by 31% compared to those who did not get this adjuvant therapy. This could at least partly explain the positive effect we observed on the prognosis of patients receiving statins.

Contrary to our and previous studies, a meta-analysis by Liang et al. [33] suggested that statin use is associated with a significant increase in the risk of developing liver injury by 18%. However, there was no clear relationship between the dose or the lipid-lowering efficacy of different statins. Nevertheless, a recent statement from an international expert panel of the European Atherosclerosis Society concludes that transient and clinically non-relevant increases in liver enzymes occur in 0.5–2% of patients taking statins, while idiosyncratic liver injury due to statins is very rare and causality is difficult to be proven [34]. The American Heart Association estimates that the risk of severe statin-related hepatotoxicity in the general population is ≈0.001% [35]. However, it is unclear whether this risk is higher in those who already have chronic liver diseases.

Statins have a large number of pleiotropic properties independent of their effect on cholesterol levels, such as improving endothelial dysfunction or having antioxidant, anti-fibrotic, anti-inflammatory, anti-proliferative, anti-angiogenic, anti-thrombotic, or immunomodulatory properties, which could positively affect liver health [36–44]. Statins have been shown to act as free radical scavengers, slowing the progression of liver cirrhosis by reducing oxidative stress reactions [45,46]. Statins could control inflammatory response in liver cirrhosis by inhibiting and eliminating the over-production of free radicals or other pernicious by-products, thereby allowing hepatic cell damage and fibrosis to be avoided in part [47,48]. Portal hypertension as a marker of irreversible liver cirrhosis can further exacerbate liver cirrhosis and establish a vicious cycle. Statins can break this circle by lowering portal pressure to improve the prognosis of liver cirrhosis [49,50]. HCC may occur as a result of chronic liver cirrhosis [51], and statins may lower the incidence of HCC by slowing the progression of liver cirrhosis. According to research ranging from the bench to bed, chronic liver cirrhosis may be a novel target for statin therapy, and combined evidence from clinical studies finally backed this up. In cardiovascular disorders, statin treatment showed significant dose-dependent effects on the prognosis of coronary artery disease (CAD) [52,53]. Similarly, statins also showed dose-dependent effects on HCC development, decompensated cirrhosis events occurrence, and progression of liver cirrhosis. Although low doses of statins are ineffective in decompensated liver cirrhosis, medium and high doses can ameliorate it. In addition, higher dose of statin has a better effect on relieving pathological progression of liver cirrhosis. In this regard, a recent meta-analysis by Facciorusso et al. [54] provided a pooled estimate of the impact of statin use on HCC occurrence. This study found a 27% decreased risk of HCC occurrence in statin users, when adjusting for several clinical and demographical parameters. This effect was more pronounced and consistent in HBV patients (56% decrease in HCC incidence) and it was found to be linearly correlated with the dose, with a 73% decreased HCC risk in patients administered a cumulative defined daily dose beyond 365. Evidence suggests lipophilic statins were associated with significantly reduced HCC (51%) compared to (27%) in hydrophilic statin users [54–56]. In addition, among the various agents studied, atorvastatin had the most substantial chemopreventive effect with a 57% reduction in HCC occurrence [54].

Our observations on the insignificant reductions (borderline) of AST and ALT by less than 10 U/L seem equally important because it is still one of the most important reasons for statin discontinuation [1–5]. We showed that chronic statin therapy reduces aminotransferase levels, which confirms that the potentially observed changes after statin initiations and/or transient dose increase may return to normal or even be reduced in comparison to baseline levels. These are compelling reasons in favour of the idea that statin therapy should not be discontinued and statins are not associated with the risk of acute liver injury [1–5].

In addition, the effect of statin on mortality rate in this study was assessed based on subgroup analysis according to geographical distribution (Asian and non-Asian countries), type of viral hepatitis (B and C), and in patients with other specific medical illnesses (HCC or cirrhosis). According to the results, we did not find any significant difference between the risk of mortality in statin users and non-users according to geographical distribution, different types of viral hepatitis, and patients with other specific medical illnesses.

According to the data presented in the study, the mortality rate between statin users and non-users in different countries shows great heterogeneity. This heterogeneity between countries is related to highly variable factors such as differences in clinical characteristics between statin users and non-users, different study designs, population samples, diseases stage, comorbidities, and different confounding covariates. For example, because of indication bias, patients who need statins were more likely to have cardiovascular disease and high mortality rate. The presence of comorbidities, rather than the use of statins, increased overall mortality.

Our meta-analysis has also some limitations. First, significant between-study heterogeneity was observed, resulting from different study designs, population samples, disease stage, and adjusted variables. Although the multivariate Cox proportional hazards model is the most commonly used method in survival analysis, the confounding covariates are different between studies. The meta-analysis also found a significant difference in follow-up time. In addition, although the meta-analysis included several large studies based on multiple registries, some of the included studies had overlap in study subjects, e.g. the Veterans Affairs data from the USA and the Hong Kong Hospital Authority data. Second, the analysis on fibrosis progression and cirrhosis development was based on only two studies, notwithstanding their size. Third, potential publication bias, indicated that potential missing publications existed. Although almost all of the included studies reported adjusted estimates that took into account major confounders, residual confounding may be source of bias. Moreover, not all studies disclosed whether statins were all used for the first time or before the study. We did not have enough data to explore the association between LDL-C reduction and hepatoprotective effects of statin therapy. Last, we were not able to evaluate the levels of aminotransferases as continuous variables to see the changes after statin therapy at different study time points (initiation of statins, dose increase/decrease, worsening of the liver function, etc.). On the other side, to the best of our knowledge, this is the first comprehensive meta-analysis clearly showing the positive prognostic effect of statins on different severe chronic liver diseases.

In conclusion, not only long-term treatment with statins seems to be safe in patients with chronic liver disease, but also it significantly improves their prognosis. These data suggest that hypercholesterolemic patients affected by chronic liver disease should always be treated with statin, being the only exception the presence of acute liver disease.

## Supplementary Material

Supplemental Material

## Data Availability

There is no raw data associated with this systematic review and meta-analysis. The authors confirm that the data supporting the findings of this study are available within the article and its supplementary materials.
