# Supplementary material for: Statin therapy in chronic viral hepatitis: a systematic review and meta-analysis of nine studies with 195,602 participants
Source: Ann Med. 2021 Jul 23;53(1):1228–43. doi: 10.1080/07853890.2021.1956686 (PMC8317925; doi:10.1080/07853890.2021.1956686)
Supplement: Supplemental Material [file IANN_A_1956686_SM5738.docx]

**Table S1- Characteristics of included studies related to mortality**

| **Authors** | **Year** | **Mortality** | | **Sample** | **Setting** | **Study Design** | **Result** | **Conclusion** |
| --- | --- | --- | --- | --- | --- | --- | --- | --- |
|  |  | **Statin**  **User** | **Statin**  **Non User** |  |  |  |  |  |
| Kim et al. [^1^](#_ENREF_1) | 2019 | N= 980 | N= 12083 | Patients with Non-Cirrhotic Chronic Hepatitis B | Health Examination Cohort of the National Health Insurance Service (NHIS) of Korea. | Retrospective cohort study | Statin users had a significantly lower liver cancer mortality in univariate analysis [HR, 0.19; 95% confidence interval (CI), 0.06–0.61 | This study showed that statin use was associated with decreased liver cancer mortality |
|  |  | 3 (0.3) | 190 (1.6) |  |  |  |  |  |
| Simon et al[^2^](#_ENREF_2) | 2019 |  |  | Nationwide population with viral hepatitis | Swedish registers | Prospective propensity score (PS)-matched cohort | 10-year mortality was significantly lower among both lipophylic (15.2% vs 7.3%, RD – 7.9 percentage points (CI: -9.6 to -6.2), and hydrophilic (16.0% vs. 11.5%; RD, −4.5 percentage points [CI, −6.0 to −3.0  Percentage points]) statin users. | In a nationwide viral hepatitis cohort, lipophilic statins were associated with significantly reduced mortality |
|  |  |  |  |  |  |  |  |  |
| Byrne et al [^3^](#_ENREF_3) | 2017 | N= 2331 | N= 5824 | HCV Monoinfected | Veterans Affairs (VA)  Medical facilities across the United States. | Cohort study | In the setting of chronic HCV alone, statin initiators had reduced death (HR, 0.42 [95% CI, .32–.54]) than nonusers | Regardless of HIV and/or chronic HCV status, statin initiators had a lower risk of ALI and death within 18 months compared with statin nonusers. |
|  |  | 75 | 385 |  |  |  |  |  |
| Byrne et al [^3^](#_ENREF_3) | 2017 | N= 1437 | N= 6249 | HIV/HCV Coinfected | Veterans Affairs (VA)  medical facilities across the United States. | Cohort study | Among HIV/HCV-coinfected patients, statin initiators had lower risks of death (HR, 0.36 [95% CI, .28–.46]) compared with statin nonusers. | Regardless of HIV and/or chronic HCV status, statin initiators had a lower risk of ALI and death within 18 months compared with statin nonusers. |
|  |  | 85 | 854 |  |  |  |  |  |
| Mohanty et al[^4^](#_ENREF_4) | 2016 | N= 1913 | N= 4897 | Veterans with Hepatitis C-related Compensated Cirrhosis | Veteran Affairs Clinical Case Registry | Retrospective cohort | Statin users had lower risk of death (HR, 0.56; 95% CI, 0.46–0.69), compared with non-users. | Based on data from the Veteran Affairs Clinical Case Registry, statin use among patients with HCV and compensated cirrhosis is associated with over 40% lower risk of cirrhosis decompensation and death. |
|  |  | 121(6.3) | 546(11.1) |  |  |  |  |  |
| Harrison et al [^5^](#_ENREF_5) | 2010 | N= 66 | N= 3004 | Patient receiving hepatitis C therapy | 118 Academic and community centers in the united states | Randomized, parallel-group, trial | Comparing statin users to patients who did not use statins, no deaths occurred in the small statin user group. | No effect of statins on death risk in HCV patients |
|  |  | 0 | 12 |  |  |  |  |  |
| Chang et al [^6^](#_ENREF_6) | 2017 | N=313 | N=292 | HBV-related cirrhosis | Taiwan National Health Insurance beneﬁciaries | Nested case-control study using a  cohort | Among patients with HBV-related cirrhosis (n 5 605), statin users had significantly lower risk of mortality (adjusted HR, 0.39; 95% CI, 0.21-0.72), | Statin use was associated with significantly lower mortality (by approximately 60%), primarily in patients  with HBV-related cirrhosis |
|  |  | 19 (6%) | 52(19%) |  |  |  |  |  |
| Chang et al [^6^](#_ENREF_6) | 2017 | N= 146 | N= 152 | HCV-related cirrhosis | Taiwan National Health Insurance beneﬁciaries | Nested case-control study using a  cohort | Among the patients with HCV-related cirrhosis (n=298) statin users showed not significantly decrease in mortality rate (12% vs. 20%; P=0.063) | No effect of statins on mortality risk in HCV-related cirrhosis |
|  |  | 18(12%) | 31(20%) |  |  |  |  |  |
| Shao et al [^7^](#_ENREF_7) | 2015 | N= 1988) | N= 18,212 | HBV carrier-related HCC Patients | National Health Insurance claims database and cancer  registry databases of The Collaboration Center of Health Information  Application, Taiwan, | Retrospective cohort | HCC patients who received palliative treatment with statin use had lower HCC-specific deaths in all stages than those who received palliative treatment without statin use (P<0.0001, 0.0002, 0.0012, and0.0002, and relative risk (RR)<0.763, 0.775, 0.839, and 0.718, for stages I–IV, respectively) | Statin-based palliative treatment in early stage HCC remarkably reduced the number of deaths. |
|  |  | 1067 (53.67) | 9223 (50.64) |  |  |  |  |  |
| Wong et al [^8^](#_ENREF_8) | 2017 | N= 2053 | N= 67,131 | Patients with chronic viral hepatitis | Hospital based database from the Hong Kong Hospital Authority | Population wide cohort study | Statin use was associated with a significant dose-dependent decrease in death (HR: 0.87; 95% CI: 0.76-0.99; P = .035) | Patients with chronic viral hepatitis who used statins have a reduced risk of liver decompensation and death compared to non-users in this propensity score weighted landmark analysis. |
|  |  | 286,  13.5 | 5292  17.2 |  |  |  |  |  |
| Hsiang et al [^9^](#_ENREF_9) | 2015 | N=934 | N=45,387 | HBV patients | Hospital  Authority database in Hong Kong | Retrospective cohort study | There was no decreased risk of death in statin users (weighted HR 0.92; 0.76-1.11, p=0.386) | No reduced death risk in HBV patients |
|  |  | 132 | 1,960 |  |  |  |  |  |

**Table S2- Characteristics of included studies related to Hepatocellular Carcinoma (HCC)**

| **Author** | **Year** | **Hepatocellular Carcinoma** | | **Sample** | **Setting** | **Study Design** | **Results** | **Conclusion** |
| --- | --- | --- | --- | --- | --- | --- | --- | --- |
|  |  | **Statin**  **User** | **Statin**  **Non User** |  |  |  |  |  |
| Goh et al [^10^](#_ENREF_10) | 2020 | N = 713 | N = 7,000 | chronic HBV-infected | Electronic databases at Samsung Medical Center. | A hospital-based  5 retrospective cohort | Statin use was associated with a lower risk of HCC (adjusted hazard ratio 12 (HR) = 0.36, 95% confidence interval (CI): 0.19-0.68) | Statin use was associated with a reduced risk of HCC development in chronic HBV-infected patients, suggesting that statin may have 20 chemopreventive role in this population |
|  |  | 30 | 672 |  |  |  |  |  |
| Chang et al [^6^](#_ENREF_6) | 2017 | N= 146 | N= 152 | HCV-related cirrhosis | Taiwan National Health Insurance beneﬁciaries | Nested case-control study using a  cohort | Among patients with HCV-related cirrhosis (n=5298), statin users showed a significantly lower risk of HCC  occurrence (6% vs. 14%; P 5 0.124) | Statin use was associated with a reduced risk of HCC development in patients with HCV-related cirrhosis. |
|  |  | 9(6%) | 22(14%) |  |  |  |  |  |
| Chang et al [^6^](#_ENREF_6) | 2017 | N= 313 | N= 292 | HBV-related cirrhosis | Taiwan National Health Insurance beneﬁciaries | Nested case-control study using a  cohort | Among patients with HBV-related cirrhosis (n=5605), statin users were not associated with a significantly lower risk of HCC occurrence (adjusted HR, 0.70; 95% CI,0.40-1.25) | No association between statin use and risk of HCC in patients with  HBV-related cirrhosis. |
|  |  | 27(9%) | 37(13%) |  |  |  |  |  |
| Mohanty et al[^4^](#_ENREF_4) | 2016 | N= 1881 | N= 4673 | Veterans with Hepatitis C-related Compensated Cirrhosis | Veteran Affairs Clinical Case Registry | Retrospective cohort | Statin use was associated with a significantly lower risk of development of HCC [HR 0.42 (95% CI 0.27, 0.64)] | Our study confirms evidence in the literature showing a decrease in HCC with statins |
|  |  | 25(1.3) | 148(3.2) |  |  |  |  |  |
| Simon et al [^11^](#_ENREF_11) | 2016 | N= 4165 | N= 4970 | Patients with HCV infection | Electronically Retrieved Cohort of HCV Infected Veterans (ERCHIVES) database | National cohort of HCV-infected veterans | Statin use was associated with a 49% reduction in incident HCC (adjusted HR 0.51, 95% CI 0.36, 0.72). | In patients with chronic HCV, statin use was associated with a dose-dependent reduction in incident cirrhosis and HCC. |
|  |  | 1.75% | 3.22% |  |  |  |  |  |
| Butt et al [^12^](#_ENREF_12) | 2015 | N=3,347 | N=3,901 | HCV-infected persons | Electronically  Retrieved Cohort of HCV Infected Veterans  (ERCHIVES). ERCHIVES | Longitudinal, national cohort of HCV infected  veterans | Statin use was significantly associated with decreased iHCC (1.2% vs. 2.6%; P < 0.01) | Statin use was associated with decreased incidence of HCC among a large cohort of HCV-positive Veterans. |
|  |  | 1.2 | 2.6 |  |  |  |  |  |
| Hsiang et al [^9^](#_ENREF_9) | 2015 | N= 934 | N= 45,387 | HBV patients, | Hospital  Authority database in Hong Kong. | Retrospective cohort study | After landmark analysis and PS weighting of baseline covariates, statin users had 32% risk reduction in HCC (weighted sub-hazard ratio (SHR) 0.68; 95% CI 0.48-0.97) compared to non-users. | In a HBV cohort, statin use is associated with reduced HCC risk by 32% after adjustment for confounders and biases. |
|  |  | 27  1.24 (0.88-1.69) | 907  0.51 (0.48-0.54) |  |  |  |  |  |

**Table S3- Characteristics of included studies related to Fibrosis**

| **Author** | **Year** | **Fibrosis** | | **Sample** | **Setting** | **Study Design** | **Result** | **Conclusion** |
| --- | --- | --- | --- | --- | --- | --- | --- | --- |
|  |  | **Statin**  **User** | **Statin**  **Non User** |  |  |  |  |  |
| Simon et al [^11^](#_ENREF_11) | 2016 | N=4165 | N=4970 | Patients with HCV infection | Electronically Retrieved Cohort of HCV Infected Veterans (ERCHIVES) database | National cohort of HCV-infected veterans | Statin use was associated with reduced risk of fibrosis progression (HR 0.66, 95% CI 0.6, 0.73; p<0.001). | There is a statistically significant inverse relationship between increasing statin dose and reduction in the HR of fibrosis progression. |
|  |  | 548  (7.6%) | 1065 (21.43%) |  |  |  |  |  |
| Simon et al[^13^](#_ENREF_13) | 2015 | N=29 | N=514 | Subjects with chronic hepatitis C (CHC) and advanced hepatic fibrosis | Multicenter  NIDDK Central Repositories | Randomized controlled trial | Fibrosis progression occurred in 3/29 (10%) statin users and 145/514 (29%) non-users. The unadjusted hazard ratio (HR) for fibrosis progression among statin users compared to non-users was 0.32 (95% CI 0.10-0.99). | Statin use is associated with a reduced risk of fibrosis progression in advanced CHC. |
|  |  | 3 (10%) | 145 (28%) |  |  |  |  |  |

**Table S4- Characteristics of included studies related to Cirrhosis**

| **Author** | **Year** | **Cirrhosis** | | **Sample** | **Setting** | **Study Design** | **Result** | **Conclusion** |
| --- | --- | --- | --- | --- | --- | --- | --- | --- |
|  |  | **Statin**  **User** | **Statin**  **Non User** |  |  |  |  |  |
| Clement et al [^14^](#_ENREF_14) | 2016 | N= 54 144 | N= 23 813 | HCV Infected patients | VA Clinical Case Registry | Retrospective cohort study | - | Statins were underutilized among veterans infected with HCV, and HIV/HCV according to previous  ATP-III guidelines. Current VA/DoD and ACC/AHA guidelines substantially expand statin recommendations and widen the gap of statin underutilization in all groups. |
|  |  | 11.1 % | 17.8 % |  |  |  |  |  |
| Clement et al [^14^](#_ENREF_14) | 2016 | N= 1534 | N= 706 | HIV/HCV Coinfected patients | VA Clinical Case Registry | Retrospective cohort study | - | Statins were underutilized among veterans infected with HCV, and HIV/HCV according to previous  ATP-III guidelines. Current VA/DoD and ACC/AHA guidelines substantially expand statin recommendations and widen the gap of statin underutilization in all groups. |
|  |  | 14.7% | 21.5% |  |  |  |  |  |
| Butt et al [^12^](#_ENREF_12) | 2015 | N=3,347 | N=3,901 | HCV-infected persons | Electronically  Retrieved Cohort of HCV Infected Veterans  (ERCHIVES). ERCHIVES | Longitudinal, national cohort | Statin use was significantly associated with decreased cirrhosis development (17.3% vs.25.2%; P < 0.001) | Statin use remained strongly associated with a lower risk of cirrhosis |
|  |  | 100 (14.0) | 1754 (25.1) |  |  |  |  |  |

**Table S5- Quality assessment of studies by Newcastle*-*Ottawa Scale (NOS)**

| **Studies** | **Selection of cohorts** | | | | **Comparability of cohorts** | **Outcome** | | | **Total Score** |
| --- | --- | --- | --- | --- | --- | --- | --- | --- | --- |
|  | Representativeness of the exposed cohort | Selection of the non-exposed cohort | Ascertainment of exposure | Demonstration that outcome of interest was not present at start of study | Comparability of cohorts on the basis of the design or analysis | Assessment of outcome | Follow-up length enough for outcomes to occur | Adequacy of cohorts follow up |  |
| Kim et al[^1^](#_ENREF_1) | ★ | ★ |  |  | ★★ | ★ | ★ | ★ | 7 |
| Mohanty et al[^4^](#_ENREF_4) | ★ | ★ | ★ | ★ | ★★ | ★ | ★ | ★ | 9 |
| Shao et al[^7^](#_ENREF_7) | ★ | ★ | ★ | ★ | ★ | ★ |  |  | 6 |
| Simon et al[^2^](#_ENREF_2) | ★ | ★ | ★ | ★ | ★★ |  |  | ★ | 7 |
| Wong et al[^8^](#_ENREF_8) | ★ | ★ | ★ | ★ | ★ | ★ |  |  | 6 |
| Hsiang et al[^9^](#_ENREF_9) | ★ | ★ |  | ★ | ★★ | ★ | ★ | ★ | 8 |
| Chang et al[^6^](#_ENREF_6) | ★ | ★ | ★ | ★ |  | ★ | ★ | ★ | 7 |
| Byrne et al[^3^](#_ENREF_3) | ★ | ★ | ★ | ★ | ★★ | ★ |  | ★ | 8 |
| Butt et al[^12^](#_ENREF_12) | ★ | ★ |  | ★ | ★ | ★ |  | ★ | 6 |
| Goh et al[^10^](#_ENREF_10) | ★ | ★ | ★ |  | ★ | ★ |  |  |  |
| Simon et al[^11^](#_ENREF_11) | ★ | ★ | ★ |  | ★★ | ★ | ★ | ★ | 8 |
| Clement et al[^14^](#_ENREF_14) | ★ | ★ |  | ★ | ★★ | ★ | ★ |  | 7 |

**Table S6- Quality assessment of studies by Jaded scale**

| **Dimension** |  | Harrison et al[^5^](#_ENREF_5) | Simon et al[^13^](#_ENREF_13) |
| --- | --- | --- | --- |
| **Randomization** | Was the study described as randomized? | 1 | 1 |
|  | The randomization was adequate? | 1 | 1 |
| **Blinding** | Was the double blinded correct? | 0 | 0 |
|  | The blinding of methodology was correct? | 0 | 0 |
| **Withdrawals and dropouts** | Losses and exclusions were described? | 1 | 1 |
| **Total Score** | | 3 | 3 |


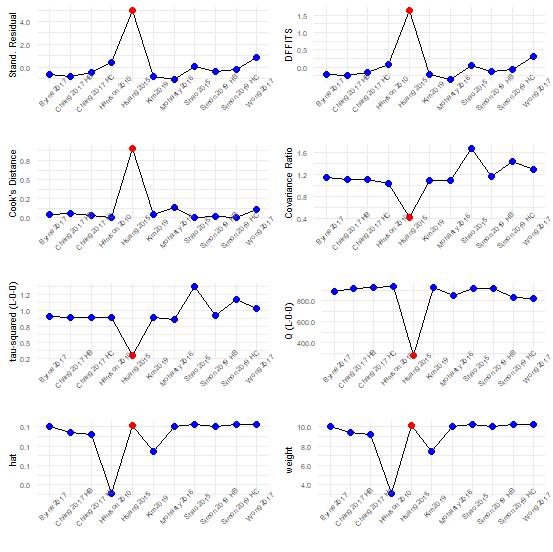


Fig S1- Influence diagnostics plot to determine outlining studies related to mortality


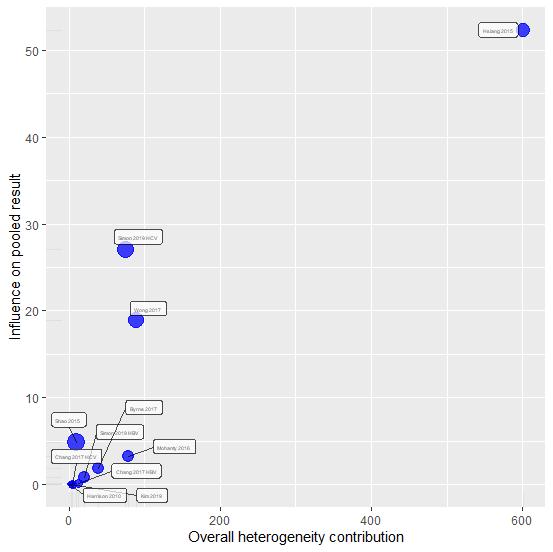


Fig S2- Baujat plot to determine heterogeneity in studies related to mortality

| 1  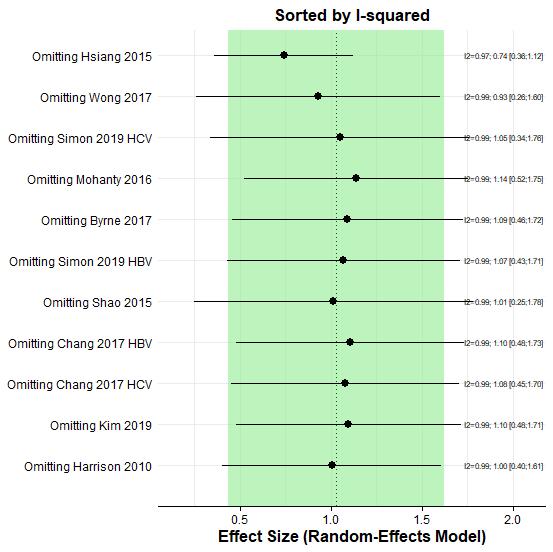 | 2  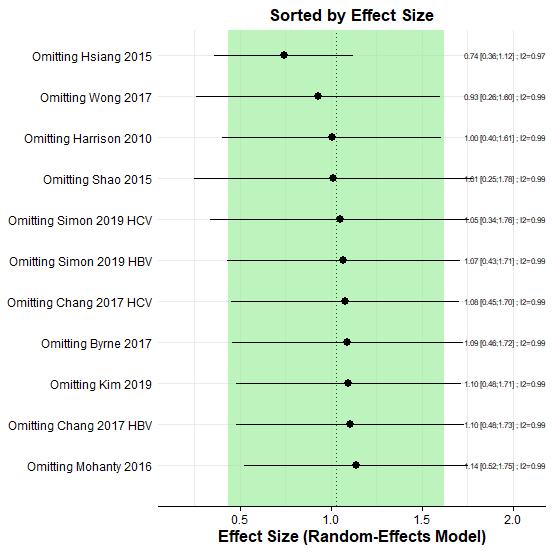 |
| --- | --- |

Fig S3- Effect size after omitting each study

1. Sorted by I-squared
2. Sorted by effect size

**References:**

1. Kim GA, Shim JJ, Lee JS, Kim BH, Kim JW, Oh CH, Oh CM, Oh IH, Park SY. Effect of Statin Use on Liver Cancer Mortality Considering Hypercholesterolemia and Obesity in Patients with Non-Cirrhotic Chronic Hepatitis B. Yonsei Medical Journal. 1203;60(12):1203-8.

2. Simon TG, Duberg A-S, Aleman S, Hagström H, Chung RT, Ludvigsson JF, editors. Lipophilic Statins and Risk of Hepatocellular Carcinoma and Mortality: A Prospective, Nationwide Population with Chronic Viral Hepatitis2019 2018: John Wiley & Sons.

3. Byrne DD, Tate JP, Forde KA, Lim JK, Goetz MB, Rimland D, Rodriguez-Barradas MC, Butt AA, Gibert CL, Brown ST, Bedimo R, Freiberg MS, Justice AC, Kostman JR, Roy JA, Lo Re V, III. Risk of acute liver injury after statin initiation by human immunodeficiency virus and chronic hepatitis c virus infection status. Clin Infect Dis. 2017;65(9):1542-50.

4. Mohanty A, Tate JP, Garcia-Tsao G. Statins are associated with a decreased risk of decompensation and death in veterans with hepatitis C–related compensated cirrhosis. Gastroenterology. 2016;150(2):430-40.

5. Harrison SA, Rossaro L, Hu KQ, Patel K, Tillmann H, Dhaliwal S, Torres DM, Koury K, Goteti VS, Noviello S, Brass CA, Albrecht JK, McHutchison JG, Sulkowski MS. Serum cholesterol and statin use predict virological response to peginterferon and ribavirin therapy. Hepatology (Baltimore, Md). 2010;52(3):864-74.

6. Chang FM, Wang YP, Lang HC, Tsai CF, Hou MC, Lee FY, Lu CL. Statins decrease the risk of decompensation in hepatitis B virus– and hepatitis C virus–related cirrhosis: A population-based study. Hepatology (Baltimore, Md). 2017;66(3):896-907.

7. Shao JY, Lee FP, Chang CL, Wu SY. Statin-Based Palliative Therapy for Hepatocellular Carcinoma. Medicine. 2015;94(42):e1801.

8. Wong JC, Chan HL, Tse YK, Yip TC, Wong VW, Wong GL. Statins reduce the risk of liver decompensation and death in chronic viral hepatitis: a propensity score weighted landmark analysis. Alimentary pharmacology & therapeutics. 2017;46(10):1001-10.

9. Hsiang JC, Wong GL, Tse YK, Wong VW, Yip TC, Chan HL. Statin and the risk of hepatocellular carcinoma and death in a hospital-based hepatitis B-infected population: A propensity score landmark analysis. Journal of Hepatology.63(5):1190-7.

10. Goh MJ, Sinn DH, Kim S, Woo SY, Cho H, Kang W, Gwak GY, Paik YH, Choi MS, Lee JH, Koh KC, Paik SW. Statin Use and the Risk of Hepatocellular Carcinoma in Patients With Chronic Hepatitis B. Hepatology (Baltimore, Md).

11. Simon TG, Bonilla H, Yan P, Chung RT, Butt AA. Atorvastatin and Fluvastatin Are Associated With Dose-Dependent Reductions in Cirrhosis and Hepatocellular Carcinoma, Among Patients With Hepatitis C Virus: Results From ERCHIVES. Hepatology (Baltimore, Md). 2016;64(1):47-57.

12. Butt AA, Yan P, Bonilla H, Abou-Samra AB, Shaikh OS, Simon TG, Chung RT, Rogal SS. Effect of addition of statins to antiviral therapy in hepatitis C virus-infected persons: Results from ERCHIVES. Hepatology (Baltimore, Md). 2015;62(2):365-74.

13. Simon TG, King LY, Zheng H, Chung RT. Statin use is associated with a reduced risk of fibrosis progression in chronic hepatitis C. Journal of hepatology. 2015;62(1):18-23.

14. Clement ME, Park LP, Navar AM, Okeke NL, Pencina MJ, Douglas PS, Naggie S. Statin Utilization and Recommendations Among HIV- and HCV-infected Veterans: A Cohort Study. Clin Infect Dis. 2016;63(3):407-13.
